# Oculomotor Behavior as a Biomarker for Differentiating Pediatric Patients With Mild Traumatic Brain Injury and Age Matched Controls

**DOI:** 10.3389/fnbeh.2020.581819

**Published:** 2020-11-12

**Authors:** Melissa Hunfalvay, Nicholas P. Murray, Claire-Marie Roberts, Ankur Tyagi, Kyle William Barclay, Frederick Robert Carrick

**Affiliations:** ^1^RightEye, LLC, Bethesda, MD, United States; ^2^Department of Kinesiology, East Carolina University, Greensville, NC, United States; ^3^Health and Social Sciences, University of the West of England, Bristol, United Kingdom; ^4^Case Western Reserve University, Cleveland, OH, United States; ^5^Centre for Mental Health Research in association with University of Cambridge, Cambridge, United Kingdom; ^6^College of Medicine, University of Central Florida, Orlando, FL, United States; ^7^MGH Institute of Health Professions, Boston, MA, United States

**Keywords:** eye-tracking, oculomotor, mTBI, concussion, pediateric case

## Abstract

**Importance:**

Children have the highest incidence of mild traumatic brain injury (mTBI) in the United States. However, mTBI, specifically pediatric patients with mTBI, are notoriously difficult to detect, and with a reliance on traditional, subjective measurements of eye movements, the subtle but key oculomotor deficits are often missed.

**Objective:**

The purpose of this project is to determine if the combined measurement of saccades, smooth pursuit, fixations and reaction time represent a biomarker for differentiating pediatric patients with mild traumatic brain injury compared to age matched controls.

**Design:**

This study used cross-sectional design. Each participant took part in a suite of tests collectively labeled the “Brain Health EyeQ” to measure saccades, smooth pursuit, fixations and reaction time.

**Participants:**

The present study recruited 231 participants – 91 clinically diagnosed with a single incident mTBI in the last 2 days as assessed by both the Glasgow Coma Scale (GCS) and Graded Symptoms Checklist (GSC), and 140 age and gender-matched controls (*n* = 165 male, *n* = 66 female, *M* age = 14.20, *SD* = 2.78).

**Results:**

One-way univariate analyses of variance examined the differences in performance on the tests between participants with mTBI and controls. ROC curve analysis examined the sensitivity and specificity of the tests. Results indicated that together, the “Brain Health EyeQ” tests were successfully able to identify participants with mTBI 75.3% of the time, providing further validation to a growing body of literature supporting the use of eye tracking technology for mTBI identification and diagnosis.

## Introduction

Mild traumatic brain injury (mTBI) occurs about once every 15 s, and the excessive frequency of these injuries costs the United States more than $77 billion dollars annually (Langlois et al., 2004; Prins et al., 2013). Ninety percent of TBI’s are classified as mild (Langlois et al., 2004; Howell et al., 2018). Clinical diagnosis of mTBI is determined by the American Congress of Rehabilitation Medicine (ACRM) definition in which “a patient with a mTBI is a person who has had a traumatically induced physiological disruption of brain function, as induced by one of the following: a loss of consciousness, any memory loss, any alteration of mental state, and/or focal neurological deficits (Bazarian et al., 2005).”

Pediatric head injury is extremely common (Schunk and Schutzman, 2012). mTBI is the most common form of head injury accounting for 75–85% of these injuries (Goldstein and Levin, 1987). Children have the highest incidence of mTBI. In the United States, mTBI occurs in 692 of 100,000 children younger than 15 years of age (Guerrero et al., 2000). Identification of pediatric mTBI differs from adult mTBI due to age-related anatomical and physiological differences, pattern of injuries based on the physical ability of the child, and difficulty in neurological evaluation in children (Araki et al., 2017). Evidence suggests that children exhibit a specific pathological response to TBI with distinct accompanying neurological symptoms (Araki et al., 2017).

An important factor contributing to this epidemic is the fact that concussions are often hard to diagnose and therefore treat (Howell et al., 2018). Most symptoms are relatively subjective and easily attributed to other conditions (Howell et al., 2018). Therefore, it is essential to build on established means of mTBI detection that are both objective and reliable (Howell et al., 2018). Currently, there are three accepted branches to mTBI diagnosis: neurological, vestibular, and oculomotor (Sussman et al., 2016). In the past, most of the oculomotor assessment was carried out subjectively through examination by clinicians, with objective measurements of symptoms, rare (Bedell and Stevenson, 2013). Research suggests that subjective measurements of eye movements are more likely to miss subtle deficits, which makes the need for reliable, objective symptom detection increasingly important. One uniquely powerful method of objectively measuring eye movements can be achieved through eye-tracking technology (Bedell and Stevenson, 2013). Eye-tracking can be used to study neurological function, oculomotor assessment and can detect abnormalities in neurocircuitry and map oculomotor dysfunction to damaged sites (Bedell and Stevenson, 2013; Lai et al., 2013; Johnson et al., 2015).

Oculomotor assessment can be further divided into the measurement of four specific types of eye movements. These include saccades, smooth pursuits, fixations, and reaction time (Land and Tatler, 2009; Leigh and Zee, 2015; Lange et al., 2018). Saccades are short and fast eye movements between fixed points; smooth pursuits use predictive tracking to stabilize moving targets, fixations are even smaller movements that focus an image on the fovea, and reaction time is the time elapsed between a sensory stimulus and the response to it (Land and Tatler, 2009; Leigh and Zee, 2015; Lange et al., 2018). Each of these different eye movements activates different parts of the brain (Wong, 2007; Møllenbach et al., 2013).

The Saccadic system focuses on the rapid movements of the fovea between fixation points (Wong, 2007). Several different brain structures are involved in the regulation of saccades, including the brain stem, pons, midbrain, and cerebral cortex (Wong, 2007). Burst neuron circuits in the brainstem are responsible for the motor signals that control the extraocular muscles in the eyes that generate saccades (Wong, 2007). There is a division of labor between the pons and the midbrain, with the pons primarily involved in generating horizontal saccades and the midbrain primarily involved in generating vertical saccades (Wong, 2007). In addition, because eye movements are closely related to cognitive behaviors in higher mammals, the cerebral cortex also plays an important role in the function of saccades both directly through the burst neuron circuit, and via the superior colliculus (Wong, 2007).

The smooth pursuit system is what allows humans to predictively track moving objects (Wong, 2007; Møllenbach et al., 2013). Because the complete smooth pursuit pathway is so complex, it is not yet completely understood (Wong, 2007). First, visual information is relayed from the striate cortex to the extrastriate areas, which contain specialized neurons that encode both eye and object movement (Wong, 2007). These extrastriate areas have connections to the brain stem, which communicates information to the cerebellum. This explains why researchers have recently found functional similarity between the saccadic and smooth pursuit systems (Wong, 2007). Pursuits are controlled primarily by a network of cortical areas, including the frontal eye field and other structures such as the superior colliculus and basal ganglia (Wong, 2007). Vertical smooth pursuits and horizon pursuits have similar pathways differing only at a spot in the pons, the y-group, and the cerebellum (Wong, 2007).

Fixations hold a stationary object on the fovea while the head is not moving and prevent the image from fading (Wong, 2007; Leigh and Zee, 2015). This process is active and involves a network of brain regions, including the parietal eye field, V5 and V5A areas, supplemental eye field, and dorsolateral prefrontal cortex (Wong, 2007). The brain stem and part of the basal ganglia and the superior colliculus are involved, although specific functions are not localized to one area. Instead, they are distributed across several (Munoz, 2002; Wong, 2007). Fixations operate like a simple negative feedback loop in which the drifting movements of the eye (not the actual target) trigger the tracking mechanism to return the eye to the target (Leigh and Zee, 2015). This behavior explains the constant microsaccades characteristic of fixations; it’s simply the gaze repeatedly returning to the target (Leigh and Zee, 2015).

Reaction time (RT) is a measure of attention (Zomeren and Brouwer, 1994). However, the applications of RT assessment are much more numerous than just measuring attention. RT has been found in numerous studies to be a marker of CNS damage and neuropathology, including mTBI (Knopman, 1991; Murtha et al., 2002; Lange et al., 2018). RT can also be used to evaluate a person’s motor skill or to determine how well they interact with their environment. RT itself is the time elapsed between the presentation of stimuli and the behavioral response (Shelton and Kumar, 2010). RT assessments can be split up into simple reaction time (SRT), choice reaction time (CRT) and discriminate reaction time (DRT) (Lange et al., 2018). SRT is a single response to a single stimulus, CRT is multiple responses to multiple stimuli and DRT is a single response to one of the multiple stimuli (Lange et al., 2018). Traditional measurements of RT often fail to account for eye-specific RT metrics, including saccadic latency, visual speed, and visual processing speed (Lange et al., 2018). Eye-tracking does measure these values, and this greater level of detail provides valuable information during RT assessment (Lange et al., 2018).

Currently, pediatric mTBIs are diagnosed using a variety of measures such as level of consciousness and length of post-traumatic amnesia (Maruta et al., 2010; Levin and Diaz-Arrastia, 2015). The Glasgow Coma Score (GCS) is commonly used to evaluate consciousness on a 13–15 scale for mTBI that accounts for a motor response, verbal response, and eye-opening ability (Arbour et al., 2016). However, the GCS is widely used but not necessarily the best measure of pediatric mTBI (Ghaffarpasand et al., 2013). Furthermore, clinicians do not usually use imagining for pediatric mTBI cases (Oakes, 2018). Therefore, The Graded Symptoms Checklist (GSC) in the Standardized Assessment of Concussion (SAC) was also used as a secondary clinical tool for measurement of mTBI as recommended by the Journal of the American Medical Association Pediatrics clinical guidelines (Adjorlolo, 2018; Lumba-Brown et al., 2018a,b). Though numerous, current methods of concussion detection are often subjective or lacking in their oculomotor components (Ventura et al., 2015). Eye tracking is capable of delivering precise and objective measurements to assist in mTBI diagnosis, and this is why it is so important to consider (Komogortsev and Karpov, 2013).

Compromised saccades, smooth pursuits, fixations, and reaction time have all been linked to mTBI. Numerous studies have found compromised saccades in patients with mTBI such as prolonged latencies and directional errors on memory-guided and antisaccades tasks and impaired self-paced saccades (Williams et al., 1997; Heitger et al., 2002; Johnson et al., 2015; DiCesare et al., 2017). Both vertical and horizontal saccades have been shown to differ in patients with mTBI, and saccades of patients with mTBI have been found especially deficient under conditions of high cognitive load (Ettenhofer et al., 2018; Hunfalvay et al., 2019). Several studies have also found deficits in smooth pursuits in patients with mTBI (Heitger et al., 2009; Hoffer et al., 2017). Patients with mTBI have been shown to have both reduced prediction and more position errors (Suh et al., 2006a,b; Armstrong, 2018). mTBI patients have also been found to have increased error and variability in gaze position and reduced smooth pursuit velocity in tracking tests (Maruta et al., 2014). Another study found that fixational errors for mTBI patients were abnormally high with evidence of increased drift, saccadic intrusions, and nystagmus (Ciuffreda et al., 2004). Though fixations do not have as much focus in current literature, this is only further reason to continue to study them. Several studies exist that consider the impact mTBI has on reaction time (MacFlynn et al., 1984; Hetherington et al., 1996; Hugenholtz et al., 1998). mTBI patients have been found to have reduced processing speed as it relates to reaction time, along with increased reaction time overall (Suh et al., 2006b; Lange et al., 2018).

Between the four eye-movements being considered, there are a plethora of studies the look at the impact of mTBI, however, none exist that consider all these components together. Nor is there much research conducted specifically on the oculomotor behavior of pediatric patients with mTBI. Nevertheless, these metrics can distinguish between mTBI and Controls, and so it stands to reason that all together, they represent a superior method of mTBI detection. Of the four factors considered, fixations especially are in need of more research. Further investigation is also necessary to determine how the four metrics interact with each other, and how the combined ability to distinguish mTBI differs from the individual capacities. The purpose of this study was to compare Brain Health EyeQ score (a composite of saccades, smooth pursuits, fixations, and reaction time) of pediatric patients with clinically diagnosed mTBI and age matched controls. A secondary purpose was to examine the reaction time responses in a choice and discriminate reaction time task.

## Materials and Methods

### Participants

Data from two-hundred and thirty-one participants were analyzed. One hundred and sixteen were clinically diagnosed as having a mTBI within 2 days of the assessment. Twenty-five of these participants were excluded (see procedure), leaving 91 total participants with mTBI. One-hundred and forty participants were age and gender matched controls. Participants were between the ages of 6–18 years (*M* = 14.20, *SD* = 2.78); 165 were males (71.4%), 66 were females (28.6%). Of the 231 participants, 68.8% were White, 3.0% were Hispanic, 0.4% were Asians, 7.4% were Black, and 20.4% opted not to report ethnicity. The groups were matched by age (see Table 1).

**TABLE 1:** Demographic data by Age and Gender.

Group (n)	Mean Age (±SD)	Females	Males
Control (140)	14.31 (2.48)	39	101
mTBI (91)	14.13 (2.97)	27	64

*n, Number; SD, Standard Deviation.*

#### Clinical Diagnosis of mTBI for Pediatric Patients

All participants had been clinically assessed by Board Certified neurologists with at least 5 years’ experience in diagnosing TBIs. Clinical diagnosis of mTBI was based on the American Congress of Rehabilitation Medicine (ACRM) definition of mTBI (Mild Traumatic Brain Injury Committee, 1993). All participants were examined using the GCS and scored between 13 and 15 on the scale. However, the GCS is widely used but not necessarily the best measure of pediatric mTBI (Ghaffarpasand et al., 2013). Furthermore, clinicians do not usually use imagining for pediatric mTBI cases (Oakes, 2018). Therefore, The Graded Symptoms Checklist (GSC) in the Standardized Assessment of Concussion (SAC) was also used as a secondary clinical tool for measurement of mTBI as recommended by the Journal of the American Medical Association Pediatrics clinical guidelines (Lumba-Brown et al., 2018a,b). Using results from Grubenhoff et al. (2010) and the American Academy of Neurology concussion grading scale pediatric patients (6–18 years of age) were evaluated as having mTBI if their GSC score was between 7.7 and 19.3 (Kelly et al., 1991; Grubenhoff et al., 2010). According to Grubenhoff et al. (2010) this yielded a 95% confidence interval for case-patients with an AAN grade 1 TBI (7.7–10.7) or grade 2 TBI (11.5–19.3) (Grubenhoff et al., 2010). Therefore, participants in the mTBI group in this study scored between 13–15 on the GCS and 7.7–19.3 on the GSC.

### Apparatus

The RightEye tests were presented on a Tobii I15 vision 15″ monitor fitted with a Tobii 90 Hz remote eye tracker and a Logitech (model Y-R0017) wireless keyboard and mouse. The participants were seated in a stationary (non-wheeled) chair that could not be adjusted in height. They sat in front of a desk in a quiet, private room. Participants’ heads were unconstrained. The accuracy of the Tobii eye tracker was 0.4° within the desired headbox of 32 cm × 21 cm at 56 cm from the screen. For standardization of testing, participants were asked to sit in front of the eye-tracking system at a distance of 56 cm (ideal positioning within the virtual headbox range of the eye tracker).

#### The Brain Health EyeQ Score (BHEQ)

The Brain Health EyeQ Score (BHEQ) includes a combination of saccade, pursuit, fixation and simple reaction time oculomotor variables. A total of 58 metrics make-up the testing model. Weights range from 0.1 to 13% across metrics. More about the individual tests and metrics can be found in published papers mentioned above (Lange et al., 2018; Hunfalvay et al., 2019; Murray et al., 2019). The metrics associated with the BHEQ score all passed reliability standards (Murray et al., 2019). Extreme gradient boosting (XGB) was used for the classification task using the Rworker GitHub repository R language version 3.5.2. The efficacy of the model was evaluated using accuracy of classification. This model also outputs the importance (weights) that each variable has on the classification accuracy. These weights were then applied to the respective metrics (variables) to calculate the percentile value of a participant compared to his/her peers within the same age group. The percentiles are then aggregated over all metrics that collapse into specific tests to calculate overall scores and percentile on that test; for example, all metrics that create circular smooth pursuit (CSP), horizontal smooth pursuit (HSP), and visual smooth pursuit (VSP) tests were used to calculate overall percentile and score for the test. Results revealed pursuit test weighting 60.93% (CSP: 8.4%; HSP: 40.4%; VSP: 12.13%); self-paced saccade test weighted 24.95% (horizontal saccade (HS): 15.57%; vertical saccade (VS): 9.38%); and fixation test contributed 14.2% weighting of the model.

#### Reaction Time Tasks

In addition to the BHEQ, we examined separately Choice Reaction Time (CRT) and Discriminate Reaction Time (DRT; see Lange et al., 2018 for further details). In brief, the CRT test, the participant viewed three stimuli and was asked to provide one of three responses. In the DRT test, the participant viewed three stimuli and was required to respond to only one stimulus.

### Procedure

Participants were recruited through RightEye clinical providers. The study was conducted in accordance with the tenets of the Declaration of Helsinki. The study protocols were approved by the Institutional Review Board of East Carolina University. The nature of the study was explained to the participants and all participants provided written consent to participate. Participants were excluded from the study they had more than a single discrete episode of mTBI (*n* = 21). Following informed consent, participants were asked to complete a prescreening questionnaire and an acuity vision screening where they were required to identify four shapes at 4 mm in diameter. If any of the prescreening questions were answered positively and any of the vision screening shapes were not correctly identified, then the participant was excluded from the study (*n* = 3). Participants were excluded from the study if they reported any of the following conditions, which may have prevented successful test calibration during the prescreening process: this included vision-related issues such as extreme tropias, phorias, static visual acuity of >20/400, nystagmus, cataracts or eyelash impediments or if they had consumed drugs or alcohol within 24 h of testing (*n* = 1) (Han et al., 2010; Holmqvist and Nystrom, 2011; Renard et al., 2015; Kooiker et al., 2016; Niehorster et al., 2017). Participants were also excluded if they were unable to pass a nine-point calibration sequence. Less than 1% of the participants fell into these categories.

Qualified participants who successfully passed the nine-point calibration sequence completed the eye-tracking tests. The calibration sequence required participants to fixate one at a time on nine points displayed on the screen. The participants had to successfully fixate on at least eight out of nine points on the screen to pass the calibration sequence. Written instructions on screen and animations were provided before each test to demonstrate appropriate behavior required in each of the tests. The testing lasted less than 5 min to complete.

### Data Analysis

The differences in the groups (control, mTBI) were analyzed on clinically verified data using JMP PRO 14.0 (SAS Institute, Cary, NC, United States). The comparison was evaluated using one-way univariate ANOVAs on the Brain Health EyeQ score, Choice Reaction Time measures (saccadic latency, visual speed, processing speed, and reaction time), and Discriminate RT measures (saccadic latency, visual speed, processing speed, and reaction time). The alpha level was set at *p* < 0.05 and Omega squared (ω^2^) was used to determine effect size. In addition, a series of ROC curve analysis were plotted for the Oculomotor variables. Significant area under the curve (AUC) with 95% confidence intervals (*p* < 0.05) was used to indicate the ability of each variable to differentiate concussed participants from non-concussed. We set our criteria for a satisfactorily accurate area under the curve (AUC) to the standard of least of 0.7 (Adjorlolo, 2018). We calculated cut-off points, sensitivity, specificity, and positive and negative predictive value (PPV and NPV, respectively) for each significant AUC. Optimal cut-off points were determined by visually assessing which score combines maximum sensitivity and specificity.

## Results

**FIGURE 1:**
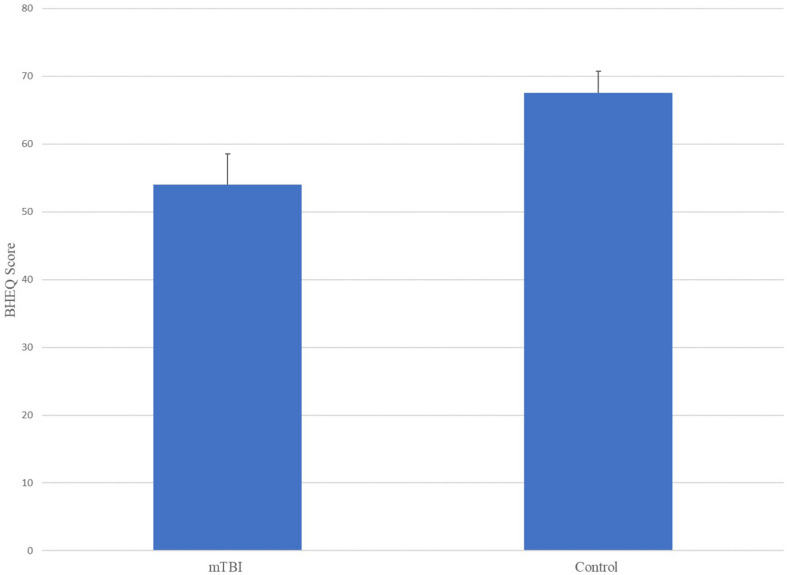
Mean Differences (with standard error) comparing BHEQ score between mTB and Control.

**FIGURE 2:**
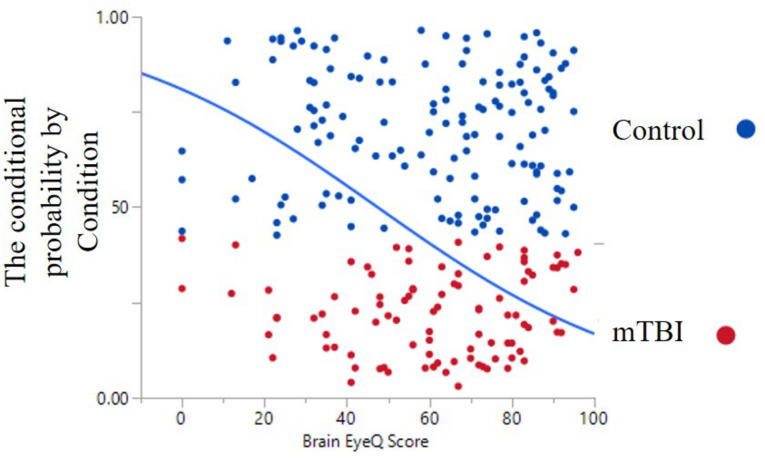
Dot Graph and probability curve for Control group (blue) and TBI group (red).

The ANOVA results for Brain Health EyeQ Score demonstrated a significant main effect for Group [*F*(1,229) = 21.906; *p* < 0.001, ω^2^ = 0.89]. The data revealed a significant difference between mTBI group (*M* = 53.98, *SD* = 20.75) and the Control group (*M* = 67.52, *SD* = 21.92; Figure 1). Further a logistic regression analysis was conducted to evaluate how well the criterion variable BHEQ predicted mTBI status (See Figure 2). The mTBI status was significantly related to the BHEQ, χ^2^ = 27.31; *p* < 0.0001, Nagelkerke *R*^2^ = 0.185.

### Choice Reaction Time (CRT)

The ANOVA results for Choice Reaction Time test demonstrated a significant main effect for Saccade Latency [*F*(1,229) = 19.53; *p* < 0.001, ω^2^ = 0.074] and processing speed [*F*(1,226) = 4.17; *p* < 0.05, ω^2^ = 0.44]. Further, we examined Visual Speed [*F*(1, 226) = 0.182; *p* = 0.670, ω^2^ = −0.003] and Reaction Time [*F*(1,224) = 0.342; *p* = 0.559, ω^2^ = .003] which demonstrated non-significant differences between Control and mTBI groups (Table 2).

**TABLE 2:** Mean and Standard Deviation for Choice Reaction Time Variables.

Group (*n*)	Saccade latency*	Processing speed*	Visual speed	Reaction time
Control	364.95 (139.83)	609.44 (227.56)	149.01 (143.20)	1123.93 (383.98)
mTBI	288.35 (109.41)	669.91 (203.61)	141.10 (126.54)	1095.77 (304.76)

*p < 0.05.

### Discriminate Reaction Time (DRT)

The ANOVA results for Discrimination Reaction Time test demonstrated a significant main effect for Saccade Latency [*F*(1,226) = 9.483; *p* < 0.01, ω^2^ = 0.35] and Processing Speed [*F*(1,219) = 15.63; *p* < 0.001, ω^2^ = 0.62]. Similar to Choice Reaction Time test, both Visual Processing Speed [*F*(1,226) = 3.544; *p* = 0.061, ω^2^ = 0.011] and Reaction Time [*F*(1,218) = 0.164; *p* = 0.686, ω^2^ = .004] did not differentiate between mTBI and Control groups in the Discriminate Reaction Time test (Table 3).

**TABLE 3:** Mean and Standard Deviation for Discriminate Reaction Time Variables.

Group (n)	Saccade latency*	Processing speed*	Visual speed	Reaction time
Control	336.81 (108.39)	379.39 (152.68)	142.32 (154.34)	856.98 (290.43)
mTBI	286.62 (136.58)	478.01 (218.24)	106.46 (117.56)	873.75 (316.35)

*p < 0.001.

### ROC Curve Analysis

**FIGURE 3:**
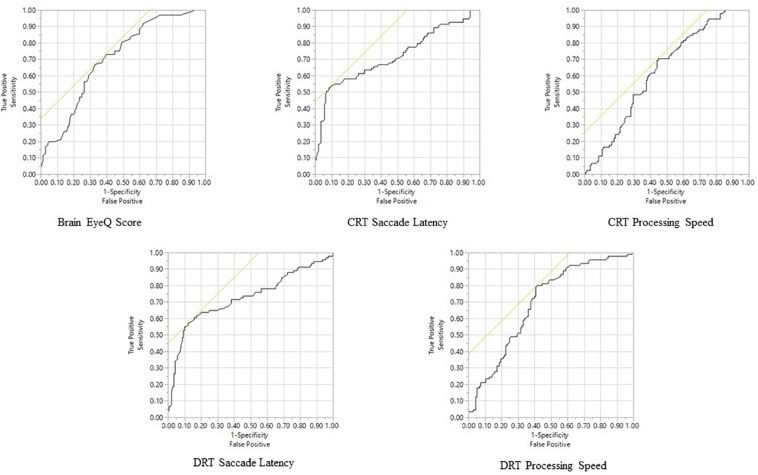
Receiver Operator Characteristic analysis predicting mTBI Status for all significant variables.

Among the RightEye variables, ROC curves were significant (*p* < 0.0001) for Brain Health EyeQ score; DRT Saccade Latency, DRT Processing Speed, CRT Saccade Latency, CRT Processing Speed CRT (Table 4 and Figure 3). ROC curves were not significant or produced low AUC score for the remaining DRT and CRT variables (Reaction Time and Visual Speed).

**TABLE 4:** Summarization of outcomes at the ROC curve analysis including: area under the curve (AUC) with standard error (S.E.), *p* values; cut-off points; sensitivity and specificity percentages; positive and negative predictive values (PPV and NPV), respectively.

Variables	AUC	S.E.	*p*	Cut-off	Sensitivity	Specificity	PPV	NPV
BHEQ	0.704*	0.00618	0.0001	63	75.3%	68.0%	73.7%	81.2%
**BHEQ subscale analysis**								
Fixation Stability	0.640	0.1346	0.0003	5.11	66.3%	67.8%	57.2%	75.4%
Horizontal Saccade Efficiency	0.560	0.0263	0.2691	7.31	59.0%	86.8%	30.6%	33.1%
Vertical Saccade Efficiency	0.597	0.210	0.0688	5.27	85.5%	81.1%	40.3%	65.1%
CSP Saccade percentage	0.68	0.273	0.0027	4.29	84.5%	71.8%	43.3%	73.3%
VSP Saccade percentage	0.55	0.018	0.2218	5.10	57.2%	11.5%	29.8%	31.5%
HSP Saccade percentage	0.42	0.17	0.698	18.45	98.6%	94.5%	40.3%	85.3%
**Reaction Time Tasks**								
DRT Saccade Latency	0.724*	0.00170	0.0039	259	58.8 %	86.4%	75.0%	75.2%
DRT Processing Speed	0.692*	0.00093	0.0004	365	73.2 %	60.7%	76.3%	76.6%
CRT Saccade Latency	0.716*	0.00138	0.0001	248	53.6%	91.4%	81.3%	74.0%
CRT Processing Speed	0.623	0.00062	0.045	578	64.9%	55.7%	70.4%	69.6%

*Represents an acceptable probability that the test differentiates mTBI from no TBI. Cut-off points (or thresholds) distinguish between a “positive” and a “negative” mTBI result and represents maximum balance between sensitivity and specificity within each test. Sensitivity represents confidence that a person has a mTBI or the true positive rate and specificity represents the accuracy of the test or the true negatives.

## Discussion

The purpose of this article was to examine the oculomotor behavior of pediatric patients with clinically diagnosed mTBI versus controls. This was done using a combination of saccade, pursuit, fixation and reaction time oculomotor variables that together made up a BHEQ Score. Results revealed a significant difference between groups, with the mTBI group showing lower (poorer) oculomotor behavior than the control group. A mean difference of 13.54% (67.52–53.98) was found. This result shows that oculomotor behavior of those with mTBI is poorer, as they scored lower than those of the control group. It also shows that the BHEQ linear combination score effectively detects such differences by examining all the major oculomotor behaviors (fixations, pursuits, and saccades). Furthermore, the BHEQ score showed a significant 0.7 AUC with a sensitivity of 75.3%. These scores indicate that the BHEQ score has a balance of sensitivity and specificity and represents the ability to discriminate whether a specific condition is present or not present. It is important to note the sensitivity and specificity are based on determining appropriate cut-off points which distinguish between a “positive” and a “negative” outcome. We utilized our data to determine these appropriate cut-scores, however, with lower cut-off scores based on minimal clinically important differences would result in better sensitivity and specificity in the measure. Furthermore, BHEQ did better overall considering AUC, *p*-value, sensitivity, and specificity of the sub-measures including pursuit test, self-paced saccade test, and fixation test and the BHEQ score has more precision in distinguishing those with mTBI and without mTBI.

It is well known that independent tests, such as saccades tests show differences between those with mTBI and those without (Hunfalvay et al., 2019). The same is true for pursuit eye movements (Suh et al., 2006b). However, to date, there has not been one combination score of all the major eye movements that a clinician can review as part of the clinical workflow to determine if there is a global oculomotor difference for a patient compared to an age matched control. One global score, one standard of reference in clinical practice, is an important benchmark for which to determine if further, more in-depth examination is required. Furthermore, the RightEye test only require 5 min to complete the test and are not impacted by acute eye fatigue during the test (Murray et al., 2019).

A secondary purpose of this article was to examine choice and discriminate reaction time tests and associated oculomotor variables between the two groups. Two variables, saccadic latency, and processing speed were found to be significantly different in both the CRT and DRT test. mTBI group had faster saccadic latency and slower processing speed than the Control group. This is consistent with past research where saccadic latency and processing speed where found to show differences between mTBI versus controls and mTBI versus athlete groups (Lange et al., 2018). Interestingly the previous research showed much larger standard deviations even with a larger sample size (*N* = 651) compared to the current research (*N* = 91). It is possible that the 10-day time limit for mTBI patients in the current study reduced the variability in results. Nevertheless, the same results were replicated. Both CRT and DRT Saccadic Latency values show a high specificity 86.4 and 91.4%, respectively. Furthermore, they showed high positive predictive values (75.0 and 81.3%). DRT and CRT Processing Speed showed high sensitivity 73.2 and 64.9%, respectively. Taken together, these metrics indicate a high predictive value, sensitivity and specificity for differentiating patients with and without mTBI. Such results further validate the use of eye movements as a biomarker for identification of mTBI. Limitations of this study include an unequal distribution of males and females in the sample populations. Past research has found conflicting evidence of gender differences in mTBI groups (Farace and Alves, 2000; Brickell et al., 2017) and future research is needed. A second limitation is that 24.7% of cases that are potentially missed. However, mTBI describes a broad term that describes a vast array of injuries and this test indicates visual motor impairment due to mTBI. Potentially, the missed cases are result from other symptoms or impairments and additional measures are needed to account for the diversity of mTBI especially in pediatric patients. A third limitation is the limited age group of pediatric patients only. Lastly, very nature of mTBI is complicated injury with completed tautology.

This study was the first to examine a combined Brain Health EyeQ score in mTBI pediatric patients. Future research should examine adults, specifically those over 65 who are the second largest group of persons who incur mTBIs and is describe as the “silent epidemic” in older adults according to Thompson et al. (2006).(Thompson et al., 2006) In conclusion, the results of this study show that (a) oculomotor behavior differs between pediatric patients with mTBI and age matched controls; (b) the BHEQ score, that combines the major categories of oculomotor behavior, differentiates pediatric patients with mTBI from controls, and (c) the CRT and DRT tests results were replicated from past research supporting the need for RT to be part of a mTBI assessment (Lange et al., 2018).

## Data Availability Statement

The raw data supporting the conclusion of this article will be made available by the authors, without undue reservation.

## Ethics Statement

The studies involving human participants were reviewed and approved by East Carolina University IRB. Written informed consent to participate in this study was provided by the participants’ legal guardian/next of kin.

## Author Contributions

All authors listed have made a substantial, direct and intellectual contribution to the work, and approved it for publication.

## Conflict of Interest

MH is Co-founder and Chief Science Officer of RightEye, headquartered in Bethesda, MD, United States. However, MH did not collect or analyze the data in this report. AT, a data scientist for RightEye, assisted in the analysis of the aggregated, deidentified data set, but was not involved in data collection. The remaining authors declare that the research was conducted in the absence of any commercial or financial relationships that could be construed as a potential conflict of interest.
